# Epigenomic diversification within the genus *Lupinus*

**DOI:** 10.1371/journal.pone.0179821

**Published:** 2017-06-22

**Authors:** Karolina Susek, Agnieszka Braszewska-Zalewska, Adam J. Bewick, Robert Hasterok, Robert J. Schmitz, Barbara Naganowska

**Affiliations:** 1 Department of Genomics, Institute of Plant Genetics, Polish Academy of Sciences, Poznan, Poland; 2 Department of Plant Anatomy and Cytology, University of Silesia in Katowice, Katowice, Poland; 3 Department of Genetics, University of Georgia, Athens, Georgia, United States of America; Saint George's University, UNITED KINGDOM

## Abstract

Deciphering the various chemical modifications of both DNA and the histone compound of chromatin not only leads to a better understanding of the genome-wide organisation of epigenetic landmarks and their impact on gene expression but may also provide some insights into the evolutionary processes. Although both histone modifications and DNA methylation have been widely investigated in various plant genomes, here we present the first study for the genus *Lupinus*. Lupins, which are members of grain legumes (pulses), are beneficial for food security, nutrition, health and the environment. In order to gain a better understanding of the epigenetic organisation of genomes in lupins we applied the immunostaining of methylated histone H3 and DNA methylation as well as whole-genome bisulfite sequencing. We revealed variations in the patterns of chromatin modifications at the chromosomal level among three crop lupins, i.e. *L*. *angustifolius* (2n = 40), *L*. *albus* (2n = 50) and *L*. *luteus* (2n = 52), and the legume model plant *Medicago truncatula* (2n = 16). Different chromosomal patterns were found depending on the specific modification, e.g. H3K4me2 was localised in the terminal parts of *L*. *angustifolius* and *M*. *truncatula* chromosomes, which is in agreement with the results that have been obtained for other species. Interestingly, in *L*. *albus* and *L*. *luteus* this modification was limited to one arm in the case of all of the chromosomes in the complement. Additionally, H3K9me2 was detected in all of the analysed species except *L*. *luteus*. DNA methylation sequencing (CG, CHG and CHH contexts) of aforementioned crop but also wild lupins such as *L*. *cosentinii* (2n = 32), *L*. *digitatus* (2n = 36), *L*. *micranthus* (2n = 52) and *L*. *pilosus* (2n = 42) supported the range of interspecific diversity. The examples of epigenetic modifications illustrate the diversity of lupin genomes and could be helpful for elucidating further epigenetic changes in the evolution of the lupin genome.

## Introduction

Epigenetic modifications of the chromatin in plant nuclear genomes are intensively studied on both the chromosome level and in the DNA/RNA sequence. However, modifications such as histone acetylation are recognised as being quite labile and can play short-term roles in genome biology [1]. A variety of modifications of chromatin can be cytologically identified and visualised and such studies have contributed to the field of epigenetics, which is the ‘study of mitotically and/or meiotically heritable changes in gene function that cannot be explained by changes in the DNA sequence’ [2]. Cytology has been used to study epigenetic phenomena among various angiosperms ranging from monocots such as *Brachiaria* [3], *Oryza* [4], *Zea* [5] to dicots like *Brassica* [6] and some legume species, including *Phaseolus vulgaris* [7], *Lathyrus sativus* and *Pisum sativum* [8].

High-throughput sequencing revolutionised epigenomic studies by enabling DNA methylation to be analysed via whole-genome bisulfite sequencing (WGBS) [9, 10] and also by revealing genome-wide interactions between DNA and proteins via whole-genome chromatin immunoprecipitation sequencing (ChIP-seq) [11, 12]. The resolution of single-base DNA methylomes has revealed that DNA methylation is ubiquitous in flowering plants, although there are a number of interesting variations [13–18].

The relevance of DNA methylation to gene duplication and the alterations in cytosine methylation that result from hybrid and/or polyploid formation can have genome-wide epigenetic consequences for the evolution of polyploids [19–21]. There is a question of whether lupins, which are legumes, are polyploids [22]. The lupin genus (*Lupinus*) encompasses about 270 annual and perennial species, which are distributed and adapted in both the Old World (generally around the Mediterranean basin) and the New World (primarily North and South America) [23, 24]. The Old World lupins are characterised by different chromosome numbers and genome sizes and an undefined ploidy levels [25].

Old World lupins consist of 12–15 annual species and include three crops–*L*. *angustifolius* (2n = 40), *L*. *albus* (2n = 50) and *L*. *luteus* (2n = 52). Genetic mapping and cross-genera macrosynteny studies between *L*. *angustifolius*, the legume model plant *Medicago truncatula* [26, 27] and *Lotus japonicus* [28] support the hypothesis that a polyploidisation event occurred early in the formation of the *Lupinus* genus. This was likely accompanied by possible structural changes in lupin genomes, such as duplications and triplications that occurred after hybridisation [29]. Molecular cytogenetic analyses have also provided insight into the multiple rearrangements that might have arisen in lupin genomes following polyploidy [30]. In contrast to Old World lupins, New World lupins encompass about 260 annual and perennial species in the genus and have a well-resolved phylogenetic relationship [31, 32]. They are also more uniform in terms of their genome structure and have a chromosome number of either 2n = 36 or 2n = 48 with a basic chromosome number of six [33].

In this study, we investigated whether histone modifications and DNA methylation are variable and whether they are associated with variations in the genome structure among closely related lupins. In particular, we studied the genome-wide distribution of chromatin modifications that are linked with either euchromatin (histone H3 dimethylation–H3K4me2) or heterochromatin (histone H3 dimethylation–H3K9me2 and DNA methylation– 5mC) [34]. The distribution of histone modifications along the chromosomes of *L*. *angustifolius*, which is used as the reference species in comparative lupin research [30, 35], was compared to the distribution in other crop relatives, i.e. *L*. *albus* and *L*. *luteus* and to *M*. *truncatula*. We conducted chromosomal immunostaining studies along with WGBS to demonstrate that this approach can be applied effectively in lupin research. These results are a prelude to further exploring epigenomic processes that may be linked with the genome evolution in this group of plants.

## Material and methods

### Plant material

The characteristics and origin of species used in this study are presented in Table 1 and S1 Fig.

**Table 1:** Characteristics of the species used in this study.

	Species	Accession	Chromosome number (2n)	Genome size^ (Mbp/2C DNA)	Country of origin
crop	*L*. *albus*	cv. ‘Boros’*	50	1137	Poland
	*L*. *angustifolius*	cv. ‘Sonet’*	40	1852	Poland
	*L*. *luteus*	cv. ‘Talar’*	52	2391	Poland
wild	*L*. *cosentinii*	98452*	32	1392	Germany
	*L*. *digitatus*	660697**	36	1343	Spain
	*L*. *micranthus*	98552*	52	960	Spain
	*L*. *pilosus*	98653*	42	1333	UK
model	*M*. *truncatula*	PI 577611**	16	931	Germany

* Polish *Lupinus* Gene Bank, Breeding Station Wiatrowo, Poznan Plant Breeders Ltd., Poland

** US Department of Agriculture, USA

^ based on [25] (considered that 1 pg = 978 Mbp)

Immunostaining was carried out on three crop lupins and *M*. *truncatula* (Table 1). Briefly, the seeds were germinated on filter paper moistened with tap water in Petri dishes in the dark at 23°C. Seedlings with about 2–3 cm long roots were treated with cold water for 12 h and fixed in either 4% formaldehyde in PBS (pH 7.3) to detect histone H3 dimethylation or in a 3:1 mixture of methanol and glacial acetic acid to detect DNA methylation. For the DNA methylation sequencing, the crop and wild lupins (Table 1) were grown in a greenhouse under controlled (watering and spray moistening, day/night control) conditions. Genomic DNA was extracted using a DNeasy Plant Mini Kit (Qiagen) following the manufacturer’s recommendations.

### Slide preparation

Root-tip meristematic cells were used as the source of the metaphase chromosomes. For slide preparation, root tips were cut off seedlings, washed in PBS (to detect histone H3 dimethylation) or a citrate buffer, pH 4.8 (to detect DNA methylation) and then digested at 37°C for 1–1.5 h with a mixture of 2.5% pectinase (Sigma-Aldrich), 2.5% cellulase Onozuka R-10 (Serva) and 2.5% pectolyase (Sigma-Aldrich) dissolved in PBS or a citrate buffer [36]. We used at least ten root meristems of each species to analyse the chromosomal distribution of epigenetic modifications using immunodetection. We analysed about ten full chromosome complements for each species, which were repeated in four timed experiments.

### Immunostaining procedures and image processing

Immunostaining was carried out as was previously described [36]. Briefly, the following rabbit monoclonal and polyclonal antibodies against modified histones and DNA were used: anti-dimethyl histone H3 at lysine 4 (1:100; Abcam, cat. no. (ab7766), anti-dimethyl histone H3 at lysine 9 (1:100; Millipore, cat. no. 07–441) and anti-5-methyl-cytosine (1:300, Abcam, cat. no. ab73938). Two secondary antibodies were also applied–Alexa Fluor 488 goat anti-rabbit IgG (Invitrogen, Molecular Probes, cat. no. A-11008) and Alexa Fluor 488 goat anti-mouse IgG (Invitrogen, Molecular Probes, cat. no. A-11001). The fluorescence of DAPI (excitation 405 nm, emission 425–475 nm) and Alexa488 (excitation 488 nm, emission 500–600 nm) was registered using an Olympus FV1000 confocal system (Olympus) equipped with an IX81 inverted microscope, a 60x Plan Apo oil-immersion objective lens (NA 1.35), a 50 mW, 405 nm diode laser and a 100 mW multi-line argon ion laser (Melles Griot BV). An axial series of 2-D fluorescence images of optical sections through the chromosomes (z-stacks) was collected using two separate photomultipliers (R6357, Hamamatsu). Image processing operations were performed with ImageJ (Fiji).

### DNA methylation sequencing and analysis

DNA was isolated from leaf tissue and MethylC-seq libraries were prepared for each species as was previously described [10, 37]. As no sequenced and assembled genome exists for *Lupinus spp*., we used *FAST*^*m*^*C*, which is a non-reference-based approach to estimate the levels of DNA methylation in the CG, CHG (H = A|C|T) and CHH sequence contexts [38]. Briefly, linear models were created for each sequence context from the DNA methylation levels that were obtained from a reference-based alignment using methylpy (actual) [39] and the levels that were estimated from the raw bisulfite sequencing reads for a diverse set of species. After controlling for genomic GC content, the actual versus estimated levels of DNA methylation showed a linear relationship [38]. Then, the raw bisulfite sequencing reads from a user-defined species can be fed into the context and taxonomic group-specific regression models to obtain an estimate of DNA methylation.

## Results

### Immunodetection of histone and DNA modifications

**Fig 1:**
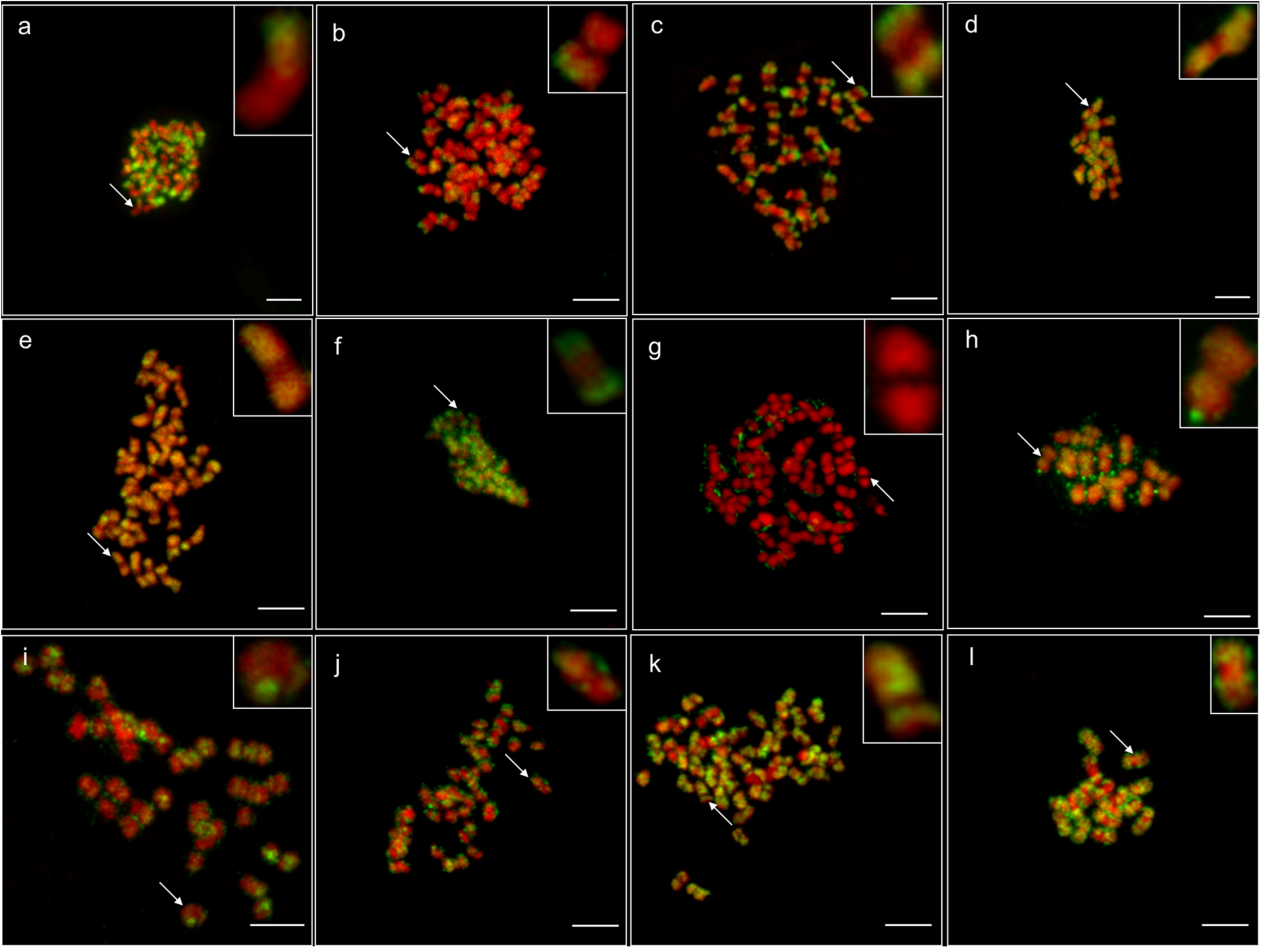
Immunodetection of histone H3 and DNA epigenetic modifications. H3K4me2: **a-d**, H3K9me2: **e-h**, 5mC: **i-l**. *L*. *angustifolius*: **c, e, i**; *L*. *albus*: **a, f, j**; *L*. *luteus*: **b, g, k**; *M*. *truncatula*: **d, h, l**. DAPI–red (false) colour, the modifications–green colour. All bars = 5 μm.

The euchromatin specific marker H3K4me2 was identified in all of the metaphase chromosomes of the analysed species in particular in the terminal parts of the chromosome arms (Fig 1A–1D). An interesting finding was that in the case of *L*. *albus* (Fig 1A) and *L*. *luteus* (Fig 1B) chromosomes, this histone modification was detected mostly within one chromosome arm. However, in *L*. *albus* the fluorescent signals on chromosomes covered most of the labelled arms, whereas they were more discrete and limited to the arm termini in *L*. *luteus*. The pattern of H3K4me2 distribution between *L*. *angustifolius* and *M*. *truncatula* (Fig 1C and 1D) was similar and was confined to the terminal regions of both chromosome arms.

Identifying the heterochromatin specific marker H3K9me2 illustrated the diverse chromosomal patterns within the analysed species. In *L*. *angustifolius* this modification was identified within the distal and proximal parts of both chromosome arms, whereas it was undetectable in the centromeres (Fig 1E). In the case of *L*. *albus*, H3K9me2 was mainly detected in the distal parts of the chromosome arms (Fig 1F), but it was not observed in *L*. *luteus* chromosomes (Fig 1G, S2F Fig). In *M*. *truncatula*, this modification was more evenly spread along the chromosomes compared to *L*. *angustifolius*, but the centromeres had a lower level of immunofluorescence (Fig 1H). We also analysed other epigenetic modifications such as H4K5ac, H4K16ac and H3K18ac (S2 Fig). The pattern of H4K5ac was invisible in the chromosomes of all of the analysed species (examples; S2A–S2C Fig). In contrast, this histone modification was observed at the high level in the interphase nuclei in *L*. *luteus* (S2A Fig) and *L*. *albus* (S2B Fig). The visualisation of H4K16ac and H3K18ac was only done for *L*. *luteus* (S2D Fig) and *L*. *albus* (S2E Fig). The acetylation of histones, similar to H3K4me2, is considered to be a euchromatin marker. H4K16ac and H3K18ac in the lupins were detected in the distal parts of the chromosome arms. The patterns of these modifications resembled those for H3K4me2.

We also studied the DNA methylation on both the chromosome and sequence levels. The chromosomal patterns of this modification in *L*. *angustifolius* were irregular and in some chromosomes encompassed their termini, whereas in the case of others, they were observed near the centromere regions (Fig 1I). Although the chromosomes of *L*. *albus* and *L*. *luteus* were also characterised by irregular patterns of 5mC, the centromeres did not have this modification in the majority of the metaphase chromosomes of these species (Fig 1J and 1K). In *M*. *truncatula* the pattern of 5mC was detected in all of the chromosomes but with a rather different distribution. Usually, two terminally and one proximally located bands could be identified within these chromosomes (Fig 1L).

### DNA methylation profiles in lupins

**Fig 2:**
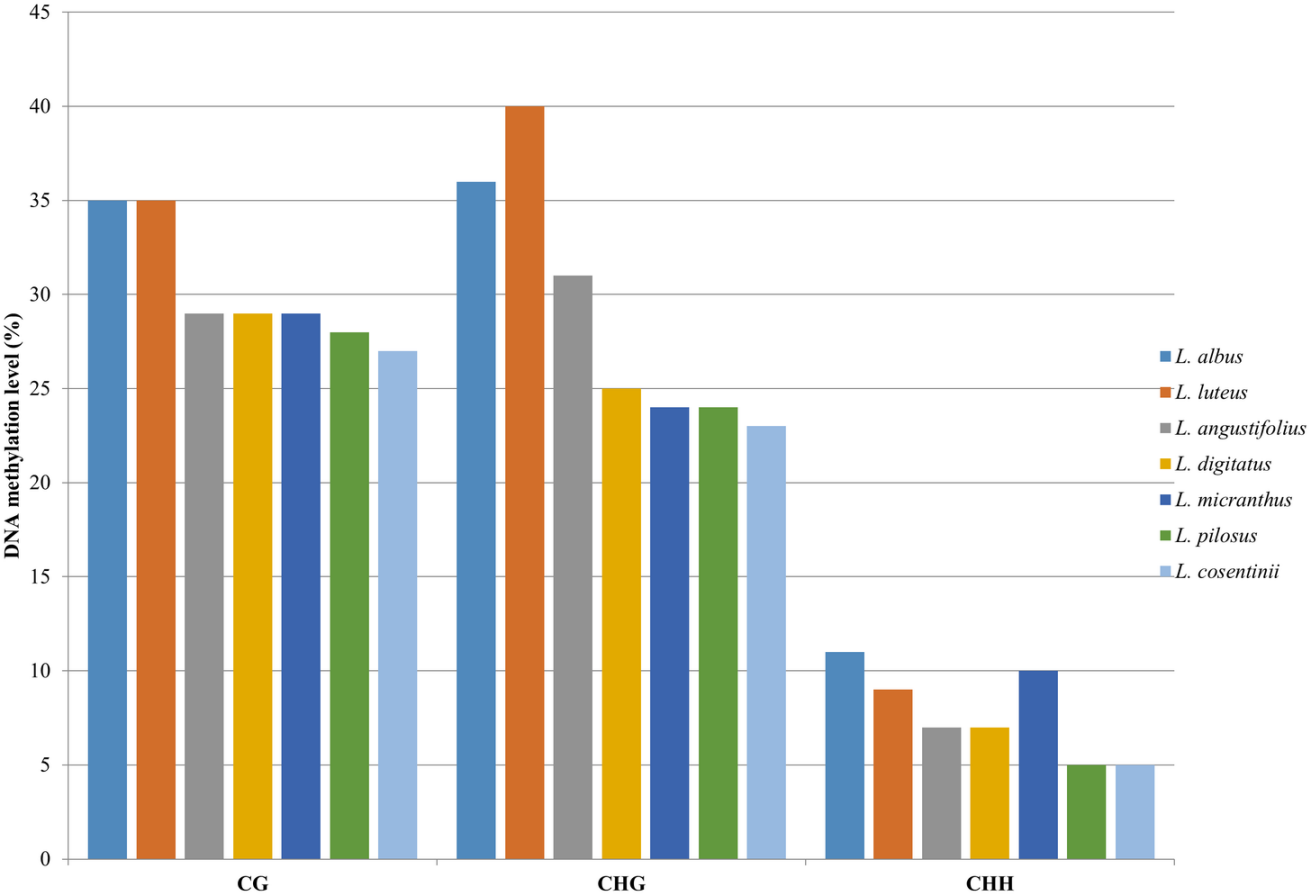
Global distribution of DNA methylation in the lupin genomes studied. The DNA profiling was analysed in the CG, CHG and CHH contexts.

*FAST*^*m*^*C* was used to address the question of the variation in DNA methylation among the three crop and four wild lupins (data obtained in this study can be found on the NCBI/GEO webpage under the accession number GSE98637). Profiling the DNA methylation revealed global differences in the patterns of DNA methylation in a symmetric (at CG sites) and non-symmetric context (CHG and CHH sites) (Fig 2). We observed that the levels of DNA methylation in the CG and CHG contexts remained approximately comparable, ranging from 27% to 35% and from 23% to 40%, respectively. Based on the level of 5mCs at the CG sites, the analysed lupins were divided into two groups according to the highest level of methylation in *L*. *albus* and *L*. *luteus* (35%) and the comparable level among *L*. *angustifolius*, *L*. *digitatus*, *L*. *micranthus* (29%), *L*. *pilosus* (28%) and *L*. *cosentinii* (27%) group. Additional analyses of the 5mCs in the CHG context illustrated that the highest level of this modification was present in *L*. *luteus* (40%) but that it remained at the lower level in *L*. *albus* (36%) and *L*. *angustifolius* (31%). The other lupins (*L*. *digitatus*, *L*. *micranthus*, *L*. *pilosus* and *L*. *cosentinii*) that were studied were ranked as the fourth common group with values ranging from 25–23%. The amount of 5mCs in the CHH context was measurably the lowest among the analysed DNA methylation-based modifications. Altogether, five groups were identified: *L*. *albus* (11%), *L*. *micranthus* (10%), *L*. *luteus* (9%), *L*. *angustifolius* and *L*. *digitatus* (7%), *L*. *pilosus* and *L*. *cosentinii* (5%). It was observed that the highest levels of 5mCs in all three nucleotide contexts were characteristic for the crop species (*L*. *albus*, *L*. *luteus*), while the lowest values were obtained for *L*. *cosentinii*, which represents the group of wild lupins.

## Discussion

H3K4me2 and H3K9me2 are well characterised histone modifications in plant genomes [40]. Chemical modifications of both the histone and DNA component of the chromatin are highly conserved among eukaryotes, although the chromosomal patterns of these modifications usually vary among species. This is mainly associated with differences in the nuclear genome composition, including the heterochromatin content, DNA content and many others variables such as stress factors or hybridisation events. Moreover, studies of epigenetic variation at the chromosomal level are of particular interest when species that are recognised as polyploids are involved [41].

Most of the data concerning the histone modifications in plants are available for *Arabidopsis thaliana* [42, 43] though some immunostaining analyses have also been performed for species such as *Vicia faba*, *Hordeum vulgare* [44], *Zea mays* [45], *Brachiaria* spp. [3] and others [46]. To date, no such studies have been carried out for *Lupinus* representatives. Our results show that the euchromatin specific marker H3K4me2 was found in the terminal parts of their chromosomes in all of the analysed species, which is in agreement with other plant species that have been studied [44]. The presence of this euchromatic marker suggests that these regions are gene-rich as was previously suggested for *L*. *angustifolius* [47] and *M*. *truncatula* [48]. Interestingly, this modification was limited to only one arm in the case of all of the chromosomes in *L*. *albus* and *L*. *luteus*, which is a finding that has not previously been reported in other plants. In the *Brachiaria* genus, some chromosomes in the complement have only one euchromatic terminal region that is methylated, which is shared by more than one chromosome pair in the tetraploids or interspecific hybrids [3]. In addition, patterns similar to those of H3K4me2 were also found for histone H3 acetylation at lysine 18 (H3K18ac) in *L*. *albus* and lysine 16 (H4K16ac) in *L*. *luteus*, which suggests that both the acetylation and methylation of the histone H3 may have some lupin-specific patterns.

The pattern of H3K9me2 modification is known to be a characteristic heterochromatin marker that is related to their nuclear genome size in many plants. This may illustrate that larger genomes are occupied by a higher amount of repetitive sequences and therefore have a lower density of genes that are distributed along the entire chromosomes that correspond to heterochromatin regions. Moreover, in species with a larger (2C > 1000 Mb) genome size, for example in *Zea mays*, the signals of H3K9me2 are evenly dispersed along the chromosome arms [45, 49]. In the case of *L*. *angustifolius* and *L*. *albus*, however, this modification was restricted to only the distal parts of the chromosome arms, which resembles the pattern that was observed for H3K4me2. It can then be assumed that heterochromatin also exists in the euchromatic chromosome blocks of lupins as was also determined for *A*. *thaliana* [50]. On the other hand, in spite of its small genome, the most dispersed and uniform chromosomal patterns of H3K9me2 were found in *M*. *truncatula*, which is in accordance with the observations of Feitoza and Guerra [51] in *Eleutherine bulbosa* but in contrast to plants with small genomes (2C < 1000 Mb), in which this histone modification preferably occupies the pericentromeric and centromeric chromosome regions [46]. Surprisingly, the dimethylation of histone H3 was not observed in the metaphase chromosomes of *L*. *luteus*, which has the largest genome size among all of the analysed species. This may imply that H3K9 methylation might have required a more complex chromatin modification system that was established during lupin evolution. These introductory studies have provided new ideas for investigating additional epigenetic markers for lupins.

The irregular occurrence of 5mC within the particular chromosomes of one species, especially in *L*. *angustifolius* and *L*. *albus*, highlights the range of genome variations. These may be associated with a qualitative (intensity of the labelling) or quantitative (percentage of labelled chromosome regions) variability of methylation-rich DNA regions in lupins. On the other hand, even in the same cell, the silencing signal can be effective in maintaining DNA methylation in one set of chromosomes but not be sufficient to silence the same sequence in a different set of chromosomes [52]. Heterogeneity concerning the distribution of 5mC signals was also detected in the tetraploid *Brachiaria ruziziensis*, in which the variation in the chromosomal distribution of this marker was observed even among cells in a single meristem. Moreover, completely hypermethylated or hypomethylated chromosomes were also observed in this species, which is similar to lupins [3]. This variation in the 5mC immunosignal distribution may result from polyploidisation processes, which are often followed by changes in DNA methylation. It is also possible that the variation in the DNA methylation in lupins may be influenced by various genome reorganisations during the polyploid evolution, especially considering that various repetitive sequences are often highly methylated and consequently affect the 5mC signal variability among lupins [53]. The diverse distribution of 5mC immunosignals among the chromosomes of different cells or even between the homologues within the same cell as well as between the arms of the same chromosome has been reported for many plant species, e.g. *Vicia faba* [54], *Haplopapus gracilis* [55] and *Brachypodium distachyon* [56]. In *M*. *truncatula*, 5mC was more evenly distributed in chromosomes and was usually restricted to the three chromosomal bands—two that were located terminally and one proximally. The pattern of 5mC in this species may reflect a specific heterochromatin block within the chromosomes. Nevertheless, apart from some *L*. *angustifolius* chromosomes, the fact that none of their chromosomes has the centromeric regions hypermethylated emphasises the importance of feature analyses of the centromere organisation in lupins.

While our knowledge of DNA methylation patterns is relatively well established in both plants and animals [57], nothing is known about it specifically in lupins. The immunofluorescence results of 5mC in the analysed *Lupinus* species encouraged us to measure the DNA methylation using WGBS. The genome-wide estimates of DNA methylation revealed that the lowest was at the CHH sites, which is precisely as was demonstrated for most of the plants that have been studied [14, 58, 59]. However, the highest level of symmetric and non-symmetric DNA methylation was always observed for two of the crop species, i.e. *L*. *luteus* and *L*. *albus*, as well as in the wild species *L*. *micranthus* in the case of the methylation at the CHH sites. This might explain why the highest amount of both genic and repetitive sequences are found in these particular species. Additionally, in *L*. *luteus*, the H3K9 marks correlate with DNA methylation [40, 52]. Interestingly, *L*. *micranthus*, which has one of the smallest known genomes among lupins, was characterised by a level of CHH methylation that was higher than in *L*. *luteus* but lower than in *L*. *albus*. This phenomenon may be associated with the specific organisation of the repetitive sequences and/or a peculiar heterochromatin condensation that could be highly packed [15, 60] and thus is accessible for maintaining the DNA methylation machinery in wild *L*. *micranthus*. Moreover, it should be emphasised that the complex polyploidisation events in lupins may be correlated with the variability in the patterns of chromatin modifications that are indicated by the complex epigenetic machinery that functions in newly formed polyploids [19]. This may mean that the patterns of DNA methylation in lupins are determined by a genome organisation. It might be also dependent on events that facilitate lupins to adapt to various environments. However, the mechanisms underlying the impact of environmental factors for establishment and maintenance of epigenetic modifications still remain elusive.

Thus, it would be worthwhile to explore the relationships between the genetic, epigenetic and phenotypic variations within this plant genus as was recently reported for *A*. *thaliana* [61].

Revealing the epigenetic organisation of chromatin in lupins can contribute to a better understanding of their complex evolutionary history, which is exemplified, for example, by the great variability in genome size and chromosome numbers. However, to address more complex research problems concerning the epigenetic organisation of lupin species, future analyses of lupins should be focussed on the DNA methylation pathways in a tissue-specific, dynamic, sequence-context-dependent and trans-generationally heritable context. Importantly, further comprehensive studies could shed some light on the role of the epigenetic modification of chromatin in lupins in their ability to adapt to a new environment.

## Supporting information

S1 Fig General map of lupin distribution. The overview of lupin geographical distribution (marked by triangles) according to Bermúdez-Torres et al. 2015. The country of origin of the studied species is indicated by a square.
(TIF)

S2 Fig Immunodetection of histone acetylation and methylation in exampled species. *L*. *luteus* H4K5ac (a), *L*. *albus* H4K5ac (b), *L*. *angustifolius* H4K5ac (c), *L*. *luteus* H4K16ac (d), *L*. *albus* H3K18ac (e), *L*. *luteus* H3K9me2 (f). All bars = 5 μm.
(TIF)

## References

[1] Springer NM, Lisch D, Li Q. Creating order from chaos: Epigenome dynamics in plants with complex genomes. Plant Cell. 2016;28(2):314–25. doi: 10.1105/tpc.15.00911 26869701 10.1105/tpc.15.00911 PMC4790878

[2] Riggs AD, Porter TN. Overview of epigenetic mechanisms. 1996 In: Epigenetic mechanisms of gene regulation. Cold Spring Harbor, NY: Cold Spring Harbor Laboratory Press 1996;29–45.

[3] de Paula CM, Souza Sobrinho F, Techio VH. Chromosomal distribution of H3K4me2, H3K9me2 and 5-methylcytosine: variations associated with polyploidy and hybridization in *Brachiaria* (Poaceae). Plant Cell Rep. 2016;35(6):1359–69. doi: 10.1007/s00299-016-1969-z 27015682 10.1007/s00299-016-1969-z

[4] Chen X, Zhou D-X. Rice epigenomics and epigenetics: challenges and opportunities. Curr Opin Plant Biol.2013;16(2):164–9. http://dx.doi.org/10.1016/j.pbi.2013.03.004. doi: 10.1016/j.pbi.2013.03.004 23562565 10.1016/j.pbi.2013.03.004

[5] Jin W, Lamb JC, Zhang W, Kolano B, Birchler JA, Jiang J. Histone modifications associated with both A and B chromosomes of maize. Chromosome Res. 2008;16(8):1203–14. doi: 10.1007/s10577-008-1269-8 18987983 10.1007/s10577-008-1269-8

[6] Braszewska-Zalewska A, Dziurlikowska A, Maluszynska J. Histone H3 methylation patterns in *Brassica nigra*, *Brassica juncea*, and *Brassica carinata* species. Genome. 2011;55(1):68–74. doi: 10.1139/G11-076 22195975 10.1139/g11-076

[7] Fonsêca A, Richard MMS, Geffroy V, Pedrosa-Harand A. Epigenetic analyses and the distribution of repetitive DNA and resistance genes reveal the complexity of common bean (*Phaseolus vulgaris* L., Fabaceae) heterochromatin. Cytogen Genome Res. 2014;143(1–3):168–78. 10.1159/000360572 24752176

[8] Neumann P, Schubert V, Fuková I, Manning JE, Houben A, Macas J. Epigenetic histone marks of extended meta-polycentric centromeres of *Lathyrus* and *Pisum* chromosomes. Front Plant Sci. 2016;7:234 doi: 10.3389/fpls.2016.00234 26973677 10.3389/fpls.2016.00234 PMC4771749

[9] Cokus SJ, Feng S, Zhang X, Chen Z, Merriman B, Haudenschild CD, et al Shotgun bisulphite sequencing of the *Arabidopsis* genome reveals DNA methylation patterning. Nature. 2008;452(7184):215–9. doi: 10.1038/nature06745 18278030 10.1038/nature06745 PMC2377394

[10] Lister R, O'Malley RC, Tonti-Filippini J, Gregory BD, Berry CC, Millar AH, et al Highly integrated single-base resolution maps of the epigenome in *Arabidopsis*. Cell. 2008;133(3):523–36. doi: 10.1016/j.cell.2008.03.029 18423832 10.1016/j.cell.2008.03.029 PMC2723732

[11] Diez CM, Roessler K, Gaut BS. Epigenetics and plant genome evolution. Curr Opin Plant Biol. 2014;18:1–8. doi: 10.1016/j.pbi.2013.11.017 24424204 10.1016/j.pbi.2013.11.017

[12] Johnson DS, Mortazavi A, Myers RM, Wold B. Genome-wide mapping of *in vivo* protein-DNA interactions. Science. 2007;316(5830):1497–502. doi: 10.1126/science.1141319 17540862 10.1126/science.1141319

[13] Schmitz RJ, He Y, Valdés-López O, Khan SM, Joshi T, Urich MA, et al Epigenome-wide inheritance of cytosine methylation variants in a recombinant inbred population. Genome Res. 2013;23(10):1663–74. doi: 10.1101/gr.152538.112 23739894 10.1101/gr.152538.112 PMC3787263

[14] Niederhuth CE, Bewick AJ, Ji L, Alabady M, Kim KD, Page JT, et al Widespread natural variation of DNA methylation within angiosperms. bioRxiv. 2016 doi: 10.1101/045880 10.1186/s13059-016-1059-0 PMC5037628 27671052

[15] Schmitz RJ, Schultz MD, Lewsey MG, O’Malley RC, Urich MA, Libiger O, et al Transgenerational epigenetic instability is a source of novel methylation variants. Science. 2011;334(6054):369 doi: 10.1126/science.1212959 21921155 10.1126/science.1212959 PMC3210014

[16] Eichten SR, Swanson-Wagner RA, Schnable JC, Waters AJ, Hermanson PJ, Liu S, et al Heritable epigenetic variation among maize inbreds. PLoS Genet. 2011;7(11):e1002372 doi: 10.1371/journal.pgen.1002372 22125494 10.1371/journal.pgen.1002372 PMC3219600

[17] Seymour DK, Koenig D, Hagmann J, Becker C, Weigel D. Evolution of DNA methylation patterns in the *Brassicaceae* is driven by differences in genome organization. PLoS Genet. 2014;10(11):e1004785 doi: 10.1371/journal.pgen.1004785 25393550 10.1371/journal.pgen.1004785 PMC4230842

[18] Li Q, Eichten SR, Hermanson PJ, Zaunbrecher VM, Song J, Wendt J, et al Genetic perturbation of the maize methylome. Plant Cell. 2014;26(12):4602–16. doi: 10.1105/tpc.114.133140 25527708 10.1105/tpc.114.133140 PMC4311211

[19] Jackson S, Chen ZJ. Genomic and expression plasticity of polyploidy. Curr Opin Plant Biol. 2010;13(2):153–9. doi: 10.1016/j.pbi.2009.11.004 20031477 10.1016/j.pbi.2009.11.004 PMC2880571

[20] Jordan WT, Schmitz RJ. The shocking consequences of hybrid epigenomes. Genome Biol. 2016;17(1):1–3. doi: 10.1186/s13059-016-0967-3 27150453 10.1186/s13059-016-0967-3 PMC4857431

[21] Rigal M, Becker C, Pélissier T, Pogorelcnik R, Devos J, Ikeda Y, et al Epigenome confrontation triggers immediate reprogramming of DNA methylation and transposon silencing in *Arabidopsis thaliana* F1 epihybrids. Proc Natl Acad Sci U S A. 2016;113(14):E2083–E92. doi: 10.1073/pnas.1600672113 27001853 10.1073/pnas.1600672113 PMC4833259

[22] Cannon SB, McKain MR, Harkess A, Nelson MN, Dash S, Deyholos MK, et al Multiple polyploidy events in the early radiation of nodulating and nonnodulating legumes. Mol Biol Evol. 2015;32(1):193–210. doi: 10.1093/molbev/msu296 25349287 10.1093/molbev/msu296 PMC4271530

[23] Drummond CS, Eastwood RJ, Miotto STS, Hughes CE. Multiple continental radiations and correlates of diversification in *Lupinus* (Leguminosae): Testing for key innovation with incomplete taxon sampling. Syst Biol. 2012;61(3):443–60. doi: 10.1093/sysbio/syr126 22228799 10.1093/sysbio/syr126 PMC3329764

[24] Gladstones JS. Distribution, origin, taxonomy, history and importance In: Gladstones JS, Atkins CA, Hamblin J, editors. Lupins as crop plants: biology, production, and utilization: CAB International; 1998 p. 1–36.

[25] Naganowska B, Wolko B, Sliwinska E, Kaczmarek Z. Nuclear DNA content variation and species relationships in the genus *Lupinus* (Fabaceae). Ann Bot. 2003;92(3):349–55. doi: 10.1093/aob/mcg145 12853281 10.1093/aob/mcg145 PMC4257507

[26] Nelson MN, Phan HT, Ellwood SR, Moolhuijzen PM, Hane J, Williams A, et al The first gene-based map of *Lupinus angustifolius* L.—location of domestication genes and conserved synteny with *Medicago truncatula*. Theor Appl Genet. 2006;113(2):225–38. doi: 10.1007/s00122-006-0288-0 16791689 10.1007/s00122-006-0288-0

[27] Kroc M, Koczyk G, Swiecicki W, Kilian A, Nelson MN. New evidence of ancestral polyploidy in the Genistoid legume *Lupinus angustifolius* L. (narrow-leafed lupin). Theor Appl Genet. 2014;127(5):1237–49. doi: 10.1007/s00122-014-2294-y 24633641 10.1007/s00122-014-2294-y

[28] Nelson MN, Moolhuijzen PM, Boersma JG, Chudy M, Lesniewska K, Bellgard M, et al Aligning a new reference genetic map of *Lupinus angustifolius* with the genome sequence of the model Legume, *Lotus japonicus*. DNA Res. 2010;17 doi: 10.1093/dnares/dsq001 20133394 10.1093/dnares/dsq001 PMC2853381

[29] Hane JK, Ming Y, Kamphuis LG, Nelson MN, Garg G, Atkins CA, et al A comprehensive draft genome sequence for lupin (*Lupinus angustifolius*), an emerging health food: Insights into plant‐microbe interactions and legume evolution. Plant Biotech J. 2016 doi: 10.1111/pbi.12615 27557478 10.1111/pbi.12615 PMC5316927

[30] Susek K, Bielski WK, Hasterok R, Naganowska B, Wolko B. A first glimpse of wild lupin karyotype variation as revealed by comparative cytogenetic mapping. Front Plant Sci. 2016;7 doi: 10.3389/fpls.2016.01152 27516770 10.3389/fpls.2016.01152 PMC4964750

[31] Nevado B, Atchison GW, Hughes CE, Filatov DA. Widespread adaptive evolution during repeated evolutionary radiations in New World lupins. Nat Commun. 2016;7:12384 doi: 10.1038/ncomms12384 27498896 10.1038/ncomms12384 PMC4979066

[32] Drummond CS, Eastwood RJ, Miotto ST, Hughes CE. Multiple continental radiations and correlates of diversification in *Lupinus* (Leguminosae): testing for key innovation with incomplete taxon sampling. Syst Biol. 2012;61(3):443–60. doi: 10.1093/sysbio/syr126 22228799 10.1093/sysbio/syr126 PMC3329764

[33] Naganowska B, Wolko B, Śliwińska E, Kaczmarek Z, Schifino-Wittmann M. 2C DNA variation and relationships among New World species of the genus *Lupinus* (Fabaceae). Plant Syst Evol. 2006;256(1):147–57.

[34] Braszewska-Zalewska AJ, Wolny EA, Smialek L, Hasterok R. Tissue-specific epigenetic modifications in root apical meristem cells of *Hordeum vulgare*. PLoS ONE. 2013;8(7):e69204 doi: 10.1371/journal.pone.0069204 23935955 10.1371/journal.pone.0069204 PMC3729647

[35] Wyrwa K, Ksiazkiewicz M, Szczepaniak A, Susek K, Podkowinski J, Naganowska B. Integration of *Lupinus angustifolius* L. (narrow-leafed lupin) genome maps and comparative mapping within legumes. Chromosome Res. 2016 doi: 10.1007/s10577-016-9526-8 27168155 10.1007/s10577-016-9526-8 PMC4969343

[36] Braszewska-Zalewska A, Bernas T, Maluszynska J. Epigenetic chromatin modifications in *Brassica* genomes. Genome. 2010;53(3):203–10. doi: 10.1139/g09-088 20237597 10.1139/g09-088

[37] Urich MA, Nery JR, Lister R, Schmitz RJ, Ecker JR. MethylC-seq library preparation for base-resolution whole-genome bisulfite sequencing. Nat Protoc. 2015;10(3):475–83. doi: 10.1038/nprot.2014.114 25692984 10.1038/nprot.2014.114 PMC4465251

[38] Bewick AJ, Hofmeister BT, Lee K, Zhang X, Hall DW, Schmitz RJ. FASTmC: A site of predictive models for nonreference-based estimations of DNA methylation. G3 (Bethesda). 2016;6(2):447–52. doi: 10.1534/g3.115.025668 26681520 10.1534/g3.115.025668 PMC4751562

[39] Schultz MD, He Y, Whitaker JW, Hariharan M, Mukamel EA, Leung D, et al Human body epigenome maps reveal noncanonical DNA methylation variation. Nature. 2015;523(7559):212–6. doi: 10.1038/nature14465 26030523 10.1038/nature14465 PMC4499021

[40] Law JA, Jacobsen SE. Establishing, maintaining and modifying DNA methylation patterns in plants and animals. Nat Rev Genet. 2010;11 doi: 10.1038/nrg2719 20142834 10.1038/nrg2719 PMC3034103

[41] Wendel JF, Jackson SA, Meyers BC, Wing RA. Evolution of plant genome architecture. Genome Biol. 2016;17(1):1–14. doi: 10.1186/s13059-016-0908-1 26926526 10.1186/s13059-016-0908-1 PMC4772531

[42] Jasencakova Z, Soppe WJJ, Meister A, Gernand D, Turner BM, Schubert I. Histone modifications in *Arabidopsis–*high methylation of H3 lysine 9 is dispensable for constitutive heterochromatin. Plant J. 2003;33(3):471–80. doi: 10.1046/j.1365-313X.2003.01638.x 12581305 10.1046/j.1365-313x.2003.01638.x

[43] Soppe WJJ, Jasencakova Z, Houben A, Kakutani T, Meister A, Huang MS, et al DNA methylation controls histone H3 lysine 9 methylation and heterochromatin assembly in *Arabidopsis*. EMBO J. 2002;21(23):6549–59. doi: 10.1093/emboj/cdf657 12456661 10.1093/emboj/cdf657 PMC136960

[44] Fuchs J, Demidov D, Houben A, Schubert I. Chromosomal histone modification patterns–from conservation to diversity. Trends Plant Sci. 2006;11(4):199–208. doi: 10.1016/j.tplants.2006.02.008 16546438 10.1016/j.tplants.2006.02.008

[45] Shi J, Dawe RK. Partitioning of the maize epigenome by the number of methyl groups on histone H3 lysines 9 and 27. Genetics. 2006;173(3):1571–83. doi: 10.1534/genetics.106.056853 16624902 10.1534/genetics.106.056853 PMC1526679

[46] Houben A, Demidov D, Gernand D, Meister A, Leach CR, Schubert I. Methylation of histone H3 in euchromatin of plant chromosomes depends on basic nuclear DNA content. Plant J. 2003;33(6):967–73. doi: 10.1046/j.1365-313X.2003.01681.x 12631322 10.1046/j.1365-313x.2003.01681.x

[47] Lesniewska K, Ksiazkiewicz M, Nelson MN, Mahe F, Ainouche A, Wolko B, et al Assignment of 3 genetic linkage groups to 3 chromosomes of narrow-leafed lupin. J Hered. 2011;102(2):228–36. doi: 10.1093/jhered/esq107 20947695 10.1093/jhered/esq107

[48] Kulikova O, Gualtieri G, Geurts R, Kim DJ, Cook D, Huguet T, et al Integration of the FISH pachytene and genetic maps of *Medicago truncatula*. Plant J. 2001;27(1):49–58. doi: 10.1046/j.1365-313x.2001.01057.x 11489182 10.1046/j.1365-313x.2001.01057.x

[49] West PT, Li Q, Ji L, Eichten SR, Song J, Vaughn MW, et al Genomic distribution of H3K9me2 and DNA methylation in a maize genome. PLoS ONE. 2014;9(8):e105267 doi: 10.1371/journal.pone.0105267 25122127 10.1371/journal.pone.0105267 PMC4133378

[50] Feng S, Jacobsen SE. Epigenetic modifications in plants: an evolutionary perspective. Curr Opin Plant Biol. 2011;14 doi: 10.1016/j.pbi.2010.12.002 21233005 10.1016/j.pbi.2010.12.002 PMC3097131

[51] Feitoza L, Guerra M. Different types of plant chromatin associated with modified histones H3 and H4 and methylated DNA. Genetica. 2011;139(3):305–14. doi: 10.1007/s10709-011-9550-8 21327493 10.1007/s10709-011-9550-8

[52] Zilberman D, Henikoff S. Silencing of transposons in plant genomes: kick them when they're down. Genome Biol. 2004;5(12):1–5. doi: 10.1186/gb-2004-5-12-249 15575975 10.1186/gb-2004-5-12-249 PMC545787

[53] Chen JZ, Ni Z. Mechanisms of genomic rearrangements and gene expression changes in plant polyploids. BioEssays: news and reviews in molecular, cellular and developmental biology. 2006;28(3):240–52. doi: 10.1002/bies.20374 16479580 10.1002/bies.20374 PMC1986666

[54] Frediani M, Giraldi E, Castiglione MR. Distribution of 5-methylcytosine-rich regions in the metaphase chromosomes of *Vicia faba*. Chromosome Res. 1996;4(2):141–6. 8785608 10.1007/BF02259707

[55] Ruffini Castiglione M, Frediani M, Venora G, Cremonini R. Cytological investigation of *Haplopappus gracilis* (Nutt.) Gray: 5-methylcytosine-rich regions, fluorochrome banding and chromatin sensitivity to DNase I digestion. Protoplasma. 2008;233(1–2):107–13. doi: 10.1007/s00709-008-0296-9 18615238 10.1007/s00709-008-0296-9

[56] Borowska N, Idziak D, Hasterok R. DNA methylation patterns of *Brachypodium distachyon* chromosomes and their alteration by 5-azacytidine treatment. Chromosome Res. 2011;19(8):955–67. doi: 10.1007/s10577-011-9243-2 22076608 10.1007/s10577-011-9243-2 PMC3228944

[57] Breiling A, Lyko F. Epigenetic regulatory functions of DNA modifications: 5-methylcytosine and beyond. Epigenetics Chromatin. 2015;8(1):1–9. doi: 10.1186/s13072-015-0016-6 26195987 10.1186/s13072-015-0016-6 PMC4507326

[58] Chen X, Ge X, Wang J, Tan C, King GJ, Liu K. Genome-wide DNA methylation profiling by modified reduced representation bisulfite sequencing in *Brassica rapa* suggests that epigenetic modifications play a key role in polyploid genome evolution. Front Plant Sci. 2015;6(836). doi: 10.3389/fpls.2015.00836 26500672 10.3389/fpls.2015.00836 PMC4598586

[59] Kim KD, El Baidouri M, Abernathy B, Iwata-Otsubo A, Chavarro C, Gonzales M, et al A Comparative epigenomic analysis of polyploidy-derived genes in soybean and common bean. Plant Physiol. 2015;168(4):1433–47. doi: 10.1104/pp.15.00408 26149573 10.1104/pp.15.00408 PMC4528746

[60] Pikaard CS, Scheid OM. Epigenetic regulation in plants. Cold Spring Harb Perspect Biol. 2014;6(12). ARTN a019315 doi: 10.1101/cshperspect.a019315 25452385 10.1101/cshperspect.a019315 PMC4292151

[61] Dubin MJ, Zhang P, Meng D, Remigereau MS, Osborne EJ, Paolo Casale F, et al DNA methylation in *Arabidopsis* has a genetic basis and shows evidence of local adaptation. Elife. 2015;4:e05255 doi: 10.7554/eLife.05255 25939354 10.7554/eLife.05255 PMC4413256

