# Supplementary figures and images for: Epigenomic diversification within the genus Lupinus
Source: PLoS One. 2017 Jun 22;12(6):e0179821. doi: 10.1371/journal.pone.0179821 (PMC5480990; doi:10.1371/journal.pone.0179821)

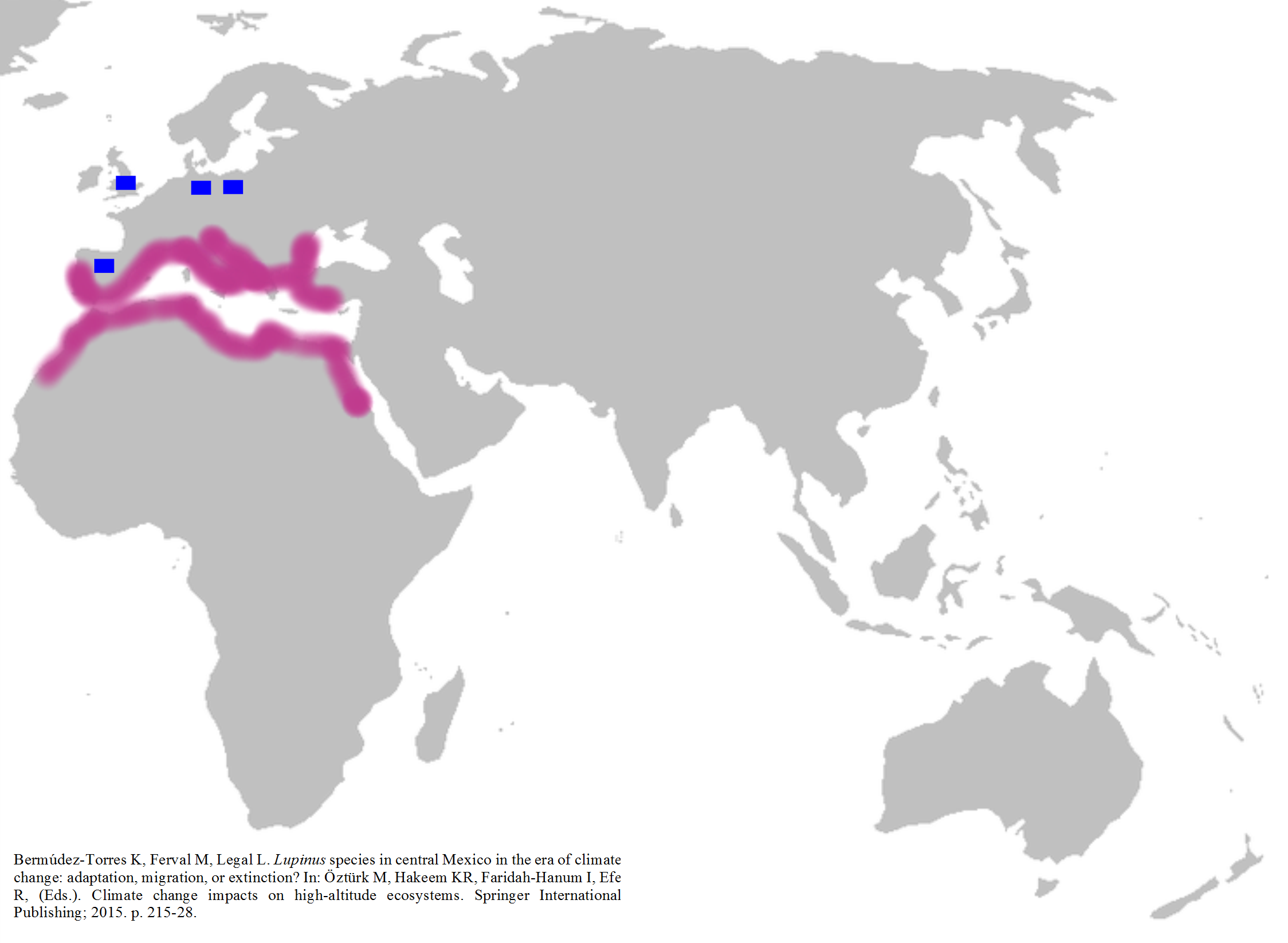

Supplement: S1 Fig — The overview of lupin geographical distribution (marked by triangles) according to Bermúdez-Torres et al. 2015. The country of origin of the studied species is indicated by a square. (TIF) [file pone.0179821.s001.tif]

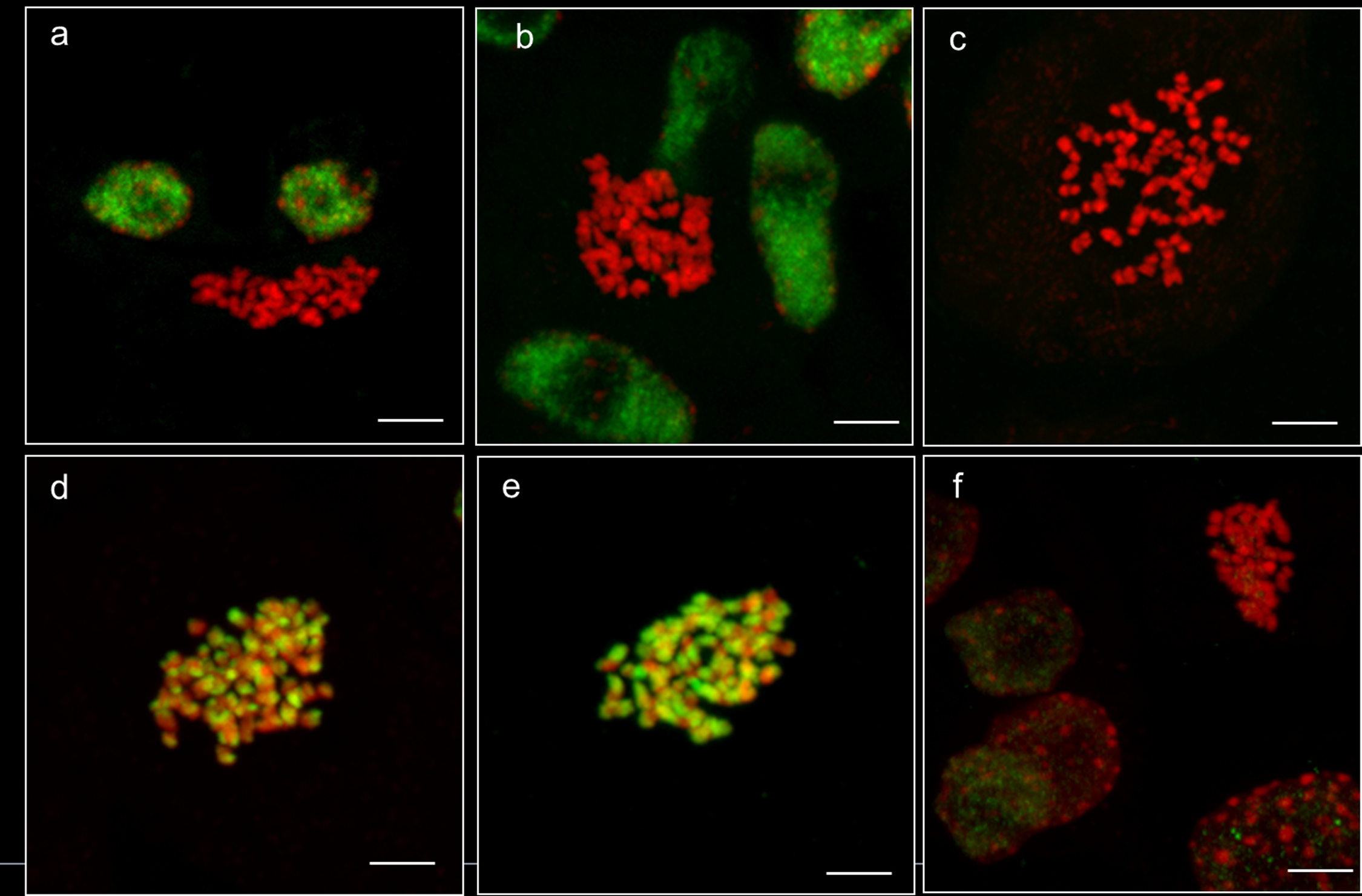

Supplement: S2 Fig — L. luteus H4K5ac (a), L. albus H4K5ac (b), L. angustifolius H4K5ac (c), L. luteus H4K16ac (d), L. albus H3K18ac (e), L. luteus H3K9me2 (f). All bars = 5 μm. (TIF) [file pone.0179821.s002.tif]
